# Total energy expenditure of elite female cheerleaders assessed by doubly labeled water

**DOI:** 10.1080/15502783.2026.2733472

**Published:** 2026-11-11

**Authors:** Mamiko Ichikawa, Yoichi Hatamoto, Aya Itoi, Naoyuki Ebine, Rie Tomiga–Takae, Kana Iwano, Yasuki Higaki

**Affiliations:** a Institute of Sports Science and Medicine, Teikyo University, Tokyo, Japan; b Faculty of Health and Sports Science, Doshisha University, Kyoto, Japan; c Faculty of Sports and Health Science, Fukuoka University, Fukuoka, Japan; d National Institute of Health and Nutrition, Osaka, Japan; e Department of Health, Sports and Nutrition, Faculty of Health and Welfare, Kobe Women’s University, Kobe, Japan

**Keywords:** Doubly labeled water, total energy expenditure, physical activity level, female athletes, cheerleading

## Abstract

**Background:**

This study aimed to quantify total energy expenditure (TEE) and physical activity level (PAL) in elite female competitive cheerleaders during the in-season period using the doubly labeled water (DLW) method.

**Methods:**

Fifteen Division 1 collegiate female competitive cheerleaders in Japan were included in the analysis. TEE was measured over seven consecutive days under free-living conditions using the DLW method. Body mass (BM) and body composition were assessed using dual-energy X-ray absorptiometry. PAL was calculated as the ratio of TEE to resting metabolic rate estimated from fat-free mass.

**Results:**

Mean TEE was 2,591 ± 440 kcal·d^−1^ (range: 1,748–3,264 kcal·d^−1^), and mean PAL was 2.21 ± 0.17 (range: 1.97–2.52). TEE showed strong positive correlations with BM (r = 0.993, *p* < 0.001) and fat-free mass (r = 0.995, *p* < 0.001). No significant change in BM was observed over the measurement period.

**Conclusions:**

This study provides the first direct assessment of TEE in elite female cheerleaders using the DLW method. The high TEE and PAL observed during the in-season period indicate that competitive cheerleading imposes substantial energetic demands comparable to those of other high-intensity sports. These findings contribute essential reference data for understanding the energy expenditure characteristics of competitive cheerleaders and may support the development of evidence-based training and health management strategies for this population.

## 1. Introduction

Competitive cheerleading is a physically demanding acrobatic team sport that combines tumbling, jumps, stunts, pyramids, and dance elements. These activities require repeated high-intensity efforts, muscular strength and power, balance, flexibility, coordination, and technical skill [1,2]. In addition, athletes perform different role-specific tasks within a team, such as flying and basing positions, which impose distinct physical demands. Flyers are generally smaller athletes performing airborne skills, whereas athletes performing basing positions repeatedly lift, throw, stabilize, and support teammates, suggesting different physiological and energetic demands. These characteristics suggest that competitive cheerleading imposes substantial physiological and energetic demands.

The popularity and competitive level of cheerleading have increased worldwide, with estimates suggesting that more than one million individuals participate annually [1,2]. As participation and competitive standards continue to increase, accurate assessment of total energy expenditure (TEE) and dietary intake is essential for estimating energy requirements and developing evidence-based nutritional strategies for competitive cheerleaders [3]. Additionally, competitive cheerleaders may be at risk of low energy availability because of inadequate energy intake relative to their training demands and may exhibit disordered eating behaviors, including restrictive and compensatory behaviors, as reported among collegiate and elite cheerleaders [3].

Several approaches are commonly used to assess energy intake or estimate energy expenditure in athletes. Dietary records provide useful information on energy and macronutrient intake but are susceptible to underreporting and cannot directly quantify free-living energy requirements [4–6]. Physiological and behavioral methods, such as heart rate monitoring [4], accelerometry [5], and activity records [6], are commonly used to estimate physical activity and energy expenditure. However, the accuracy of wearable devices varies according to activity type and movement characteristics, and accelerometer-based estimates of energy expenditure are limited for complex movement patterns [7,8]. These limitations may be particularly relevant in competitive cheerleading because routines involve repeated tumbling, stunting, partner lifting, resistance-type actions, and close physical contact that may not be adequately captured by wearable methods.

The doubly labeled water (DLW) method provides an objective assessment of TEE under free-living conditions and is regarded as the criterion method for this purpose [9,10]. In addition, physical activity level (PAL), calculated as the ratio of TEE to resting metabolic rate (RMR), is widely used for evaluating habitual physical activity and estimating energy requirements [11]. Recent studies have applied the DLW method to quantify energy expenditure in various athletic populations, including female soccer, football, and rugby players [12–14]. Despite these advances, no previous study has objectively quantified TEE and PAL in elite female competitive cheerleaders using the DLW method under free-living conditions. This gap limits the development of evidence-based nutritional strategies for elite competitive cheerleaders.

Therefore, the primary purpose of the present study was to objectively quantify TEE and PAL in elite female competitive cheerleaders during a typical in-season training period using the DLW method. A secondary purpose was to describe energy and macronutrient intake assessed by dietary records in relation to measured TEE. In addition, exploratory analyzes were conducted to examine position-specific differences in TEE, PAL, body composition, and dietary intake.

## 2. Methods

### 2.1. Participants

A total of 16 elite female cheerleaders from Division 1 of the Japan Cheerleading Association voluntarily participated in this study. All participants belonged to the same college cheerleading club. The team recently won the All-Japan Championships (2017, 2018, 2020, 2022, 2023, and 2024) and the World Championships (2019 and 2023). None of the participants had a history of diabetes or other metabolic disorders. All A-team players (*N* = 16) attended an information session and were informed about the benefits, experimental procedures, and associated risks. Those interested in participating in this study provided written informed consent.

One participant withdrew because of an injury before the measurements; therefore, data from 15 participants (age: 20.1 ± 1.1 years, range 18–22 years) were included in the final analyzes. The remaining 15 participants completed the study without injury. The study was conducted in accordance with the Declaration of Helsinki and approved by the Ethics Committee of Teikyo University (approval no. 21-112-3). Measurements were taken during the autumn competitive season of 2021, specifically during a moderate training period approximately 2 months before the competition.

### 2.2. Positional classification

Participants were classified according to their primary competitive role as either flyers or athletes performing middle, base, or spotter positions (MBS). Flyers were athletes who primarily performed airborne skills during stunts and pyramids, whereas the MBS group included athletes responsible for lifting, throwing, stabilizing, supporting, or catching flyers. Because middle, base, and spotter positions share the primary role of supporting flyers and the number of participants in each individual position was limited, these positions were combined into a single MBS group for the position-specific analyzes. Each participant’s primary position was determined based on the role regularly performed during team training and competition.

### 2.3. Overview of the study design

Figure 1 shows an overview of the measurement protocol. TEE was assessed over 7 consecutive days. During the measurement period, dietary intake was recorded on three nonconsecutive days. Body mass (BM), body composition, and blood samples were assessed after a 10-h overnight fast on day 0 (isotope administration day) and day 7.

**Figure 1.**
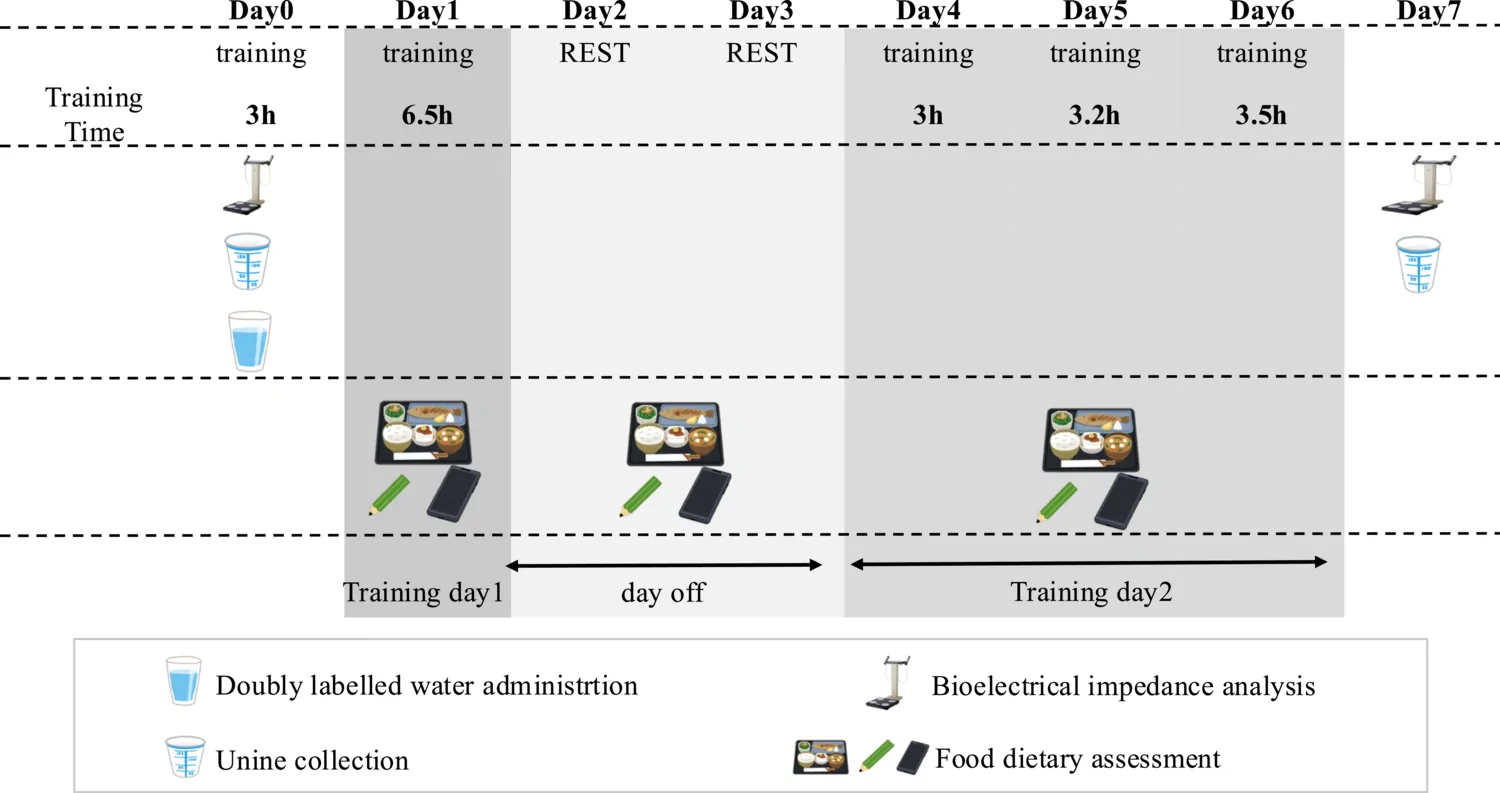
Study design and overview of the 7-day doubly labeled water measurement protocol.Total energy expenditure (TEE) was assessed over a 7-day period using the doubly labeled water method under free living conditions. Measurements were conducted from day 0 to day 7. Day 2 and day 3 were designated as rest days, during which no scheduled team training was performed. Training days included day 0, day 1, and day 4–6. Dietary intake was assessed on three non-consecutive days, including one training day before the rest period (training day 1), one rest day, and one training day after the rest period (training day 2). Body composition was assessed using bioelectrical impedance analysis, and urine samples were collected according to the DLW protocol.

Participants were instructed to refrain from alcohol and stimulant intake for at least 10 h prior to each laboratory visit. Additional details regarding body composition and blood sampling procedures are provided in the Supplementary Material.

### 2.4. Total energy expenditure (TEE)

TEE was determined using the DLW method, widely regarded as the gold standard for quantifying free-living energy expenditure owing to its high validity and reliability [15–18].

Following baseline urine collection, participants received an oral dose of stable isotope-labeled water containing ^2^H₂O (99.8 atom%; Taiyo Nippon Sanso, Tokyo, Japan) and H₂^18^O (10.0 atom%; Taiyo Nippon Sanso, Tokyo, Japan). The administered dose was 0.12 g·kg^−1^ of estimated total body water (TBW) as ^2^H₂O and 2.5 g·kg^−1^ of estimated TBW as H₂^18^O. Estimated TBW was assumed to be 60% of initial BM. The isotopes were diluted in bottled drinking water and consumed in a single dose. The dosing bottle was rinsed twice with approximately 30 mL of water, which was also consumed. Urine samples were collected at baseline and at 3 and 4 h postdosing on day 0. The 3-h sample was obtained to confirm that at least one void had occurred. Participants collected urine samples according to the study protocol and recorded the collection time for each sample. The urine samples were returned to the research staff at each study visit and stored at −20 °C until isotope analysis. During the equilibration period, participants consumed 555 mL of water. This intake was recorded and incorporated into TBW calculations.

TBW was calculated from ^2^H enrichment in the 4-h urine sample relative to baseline using a correction factor of 1.041 for nonaqueous hydrogen exchange [16,18–20]. Stable isotope enrichments were measured by isotope ratio mass spectrometry (IRMS) as previously described [16,20].

Carbon dioxide production (rCO₂; mol·d^−1^) was calculated from isotope elimination rates using the two-point method [10]

0.4554×TBW(mol)×(1.007×kO−1.043×kD)

The elimination rate constants for ^2^H and ^18^O (kD and kO, respectively) were derived from initial and final urine samples.

TEE was calculated using the modified Weir equation [21]:

$$\mathrm{TEE}\;(\mathrm{kca}\;d^{-1})=1.10\times {\mathrm{rCO}}_{2}+3.94\times ({\mathrm{rCO}}_{2}/\mathrm{RQ}),$$

where rCO₂ is the rate of carbon dioxide production (mol·d^−1^) derived from DLW, and RQ is the respiratory quotient assumed to be equal to the food quotient (FQ).

Dietary intake was assessed using 3-day self-reported dietary records and analyzed using commercially available software (WELLNESS21 Top Business System Co., Okayama, Japan). Dietary records were reviewed with each participant by trained registered dietitians to confirm portion sizes, clarify missing or ambiguous entries, and verify food items using written records and smartphone photographs. The food quotient was calculated as follows [22]:

FQ=(protein×0.81)+(fat×0.71)+(carbohydrate×1.00)

### 2.5. Physical activity level (PAL) and physical activity energy expenditure (PAEE)

PAL was calculated as TEE divided by resting metabolic rate (RMR). PAL is a dimensionless index derived from TEE relative to resting metabolic rate and allows comparison of habitual PAL between individuals while accounting for differences in body size and body composition [11]. Physical activity energy expenditure (PAEE) was estimated as:

PAEE=(TEE×0.90)−RMR

assuming a thermic effect of food equivalent to 10% of TEE [11].

### 2.6. Resting metabolic rate (RMR)

RMR was estimated using a prediction equation developed and validated for Japanese female athletes [23]:

$$\mathrm{RMR}\;(\mathrm{kcal}\cdot d^{-1})=27.5\times \mathrm{fat}-\mathrm{free}\;\mathrm{mass}\;(\mathrm{kg})+5$$

### 2.7. Assessment of energy and macronutrient intake (EI)

Three-day dietary records were collected, including one training day (full-day or half-day), one typical weekday, and one weekend rest day, to reflect the typical in-season routine. Food intake was assessed using self-reported written dietary records and smartphone photographs. All dietary records were reviewed by trained registered dietitians, and participants were interviewed when necessary to clarify missing or ambiguous information, confirm food items, and estimate portion sizes.

Dietary intake data were collected during the TEE measurement period and adjusted using a weighted average method to estimate habitual intake. Specifically, data were averaged from one weekday training day (training day 1: full- or half-day training [Day 1]; training day 2: one day selected from [Day 4, 5, or 6]) and one weekend rest day selected from [Day 2 or 3].

### 2.8. Body composition and bone mineral density (BMD)

Whole-body dual-energy X-ray absorptiometry (DXA; KONICA MINOLTA CS-7, version 1.33, Japan) was performed by the same trained operator in accordance with established best-practice procedures [24]. DXA was used to measure the total BM, body fat percentage, fat mass (FM), fat-free mass (FFM), BMD, bone mineral content, and t-scores of each player.

BM, body composition, and height were measured on the morning of day 0 and day 7, as well as on the day of the DXA measurement. Body composition (FM [kg] and FFM [kg]) was evaluated using bioelectrical impedance analysis (BIA) under standardized conditions (after > 10 h of overnight fasting) using a BIA device (TANITA, TMC-780A-*N*, Japan).

### 2.9. Statistical analysis

Because this was an observational descriptive study designed to quantify free-living TEE and PAL in a rare cohort of elite female competitive cheerleaders, no formal a priori sample size calculation was performed. Instead, the sample size was determined using an available-population approach, whereby all eligible A-team athletes during the measurement period were invited to participate. The final sample (*n* = 15) was comparable to those of previous doubly labeled water studies in elite female athletes, including professional female football players (*n* = 15) [13] and international-standard female soccer players (*n* = 24) [12]. Given the descriptive nature of the study and the limited sample size, all position-specific comparisons were considered exploratory and interpreted with caution.

Differences in BM and body composition between day 0 and day 7 were analyzed using a paired t-test. Furthermore, 95% confidence intervals for the mean differences were calculated. The relationships between TEE and variables such as BM, body composition (FFM), and total energy intake (TEI) were assessed using Pearson’s correlation coefficients. Statistical analyzes were conducted using GraphPad Prism (version 7.02; San Diego, CA, USA). Data are presented as means ± standard deviation.

## 3. Results

Table 1 presents the baseline characteristics and body composition of the study participants. Table 2 presents the changes in BM over the 7-day DLW measurement period.

**Table 1.** Baseline characteristics of division 1 female cheerleaders.

	All	Flyer	MBS
Position	*n* = 15	*n* = 4	*n* = 11
Age (years)	20.1 ± 1.1	19.3 ± 1.0	20.5 ± 1.0
Height (cm)	157.2 ± 5.6	150.0 ± 0.8	159.8 ± 4.0
Body mass (kg)	53.6 ± 7.5	43.6 ± 5.8	57.3 ± 3.7
Body mass index (kg.m^−2^)	21.6 ± 2.2	21.6 ± 2.3	22.4 ± 1.3
Fat mass (kg)	11.2 ± 2.5	9.3 ± 2.5	11.9 ± 2.3
Fat-free mass (kg)	42.4 ± 5.6	34.2 ± 3.5	45.3 ± 2.1
Body fat percentage (%)	20.8 ± 2.8	21.2 ± 3.3	20.7 ± 2.8
Whole body bone mineral content (g)	2135 ± 243	1846 ± 62	2241 ± 189
Whole body bone mineral density (g·cm^−2^)	1.14 ± 0.07	1.09 ± 0.03	1.15 ± 0.07
Whole body Z-score (%)	1.2 ± 1.3	0.4 ± 0.6	1.5 ± 1.3
Predicted RMR (kcal·d^−1^)	1170 ± 155	946 ± 97	1252 ± 58

Mean ± SD Values are presented as mean ± standard deviation. Player characteristics are shown for the total sample and according to playing position (flyers and MBS). MBS indicates middle (side base), base, and spotter positions. Body mass and stature were measured separately, and body mass index (BMI) was calculated as body mass divided by height squared. Fat mass (FM), fat-free mass (FFM), percent body fat, whole-body bone mineral content (BMC), bone mineral density (BMD), and Z-scores were assessed using dual-energy X-ray absorptiometry (DXA). Resting metabolic rate (RMR) was predicted using the equation for Japanese female athletes:

RMR (kcal·d^−1^) = 27.5 × FFM (kg) + 5 (Taguchi et al., 2011).

The initial and final BM were not significantly different (53.3 ± 7.4 kg vs. 53.7 ± 7.4 kg, respectively; *p* = 0.879).

**Table 2.** Changes in body mass and body composition over the 7-day measurement period.

	Pre	Post	
Body mass (kg)	53.3 ± 7.4	53.7 ± 7.4	*p* = 0.879
Fat mass (kg)	10.1 ± 2.9	10.5 ± 2.9	*p* = 0.690
Fat-free mass (kg)	43.2 ± 5.0	43.2 ± 5.1	*p* = 0.994
Body fat percentage (%)	18.6 ± 3.7	19.3 ± 3.5	*p* = 0.602

Mean ± SD Values are presented as mean ± standard deviation (*n* = 15). Pre- and post-measurements were obtained on days 0 and 7 of the doubly labeled water measurement period. Differences between pre- and post-measurements were analyzed using paired t-tests. Body mass was measured using a calibrated digital scale. Fat mass (FM), fat-free mass (FFM), and body fat percentage were assessed using bioelectrical impedance analysis (BIA).

### 3.1. TEE and PAL

The mean TEE over the 7-day period was 2,591 ± 440 kcal·d^−1^ (range: 1,748–3,264 kcal·d^−1^), corresponding to 48.3 ± 4.2 kcal·kg^−1^ BM·d^−1^ (range: 41.4–55.2 kcal·kg^−1^ BM·d^−1^) and 61.0 ± 4.6 kcal·kg^−1^ FFM·d^−1^ (range: 54.3–69.5 kcal·kg^−1^ FFM·d^−1^). The mean PAL was 2.21 ± 0.17 (range: 1.97–2.52).

Individual data points by positional group are shown in Figure 2. Athletes were classified as flyers or MBS (middle, base, and spotter).

**Figure 2.**
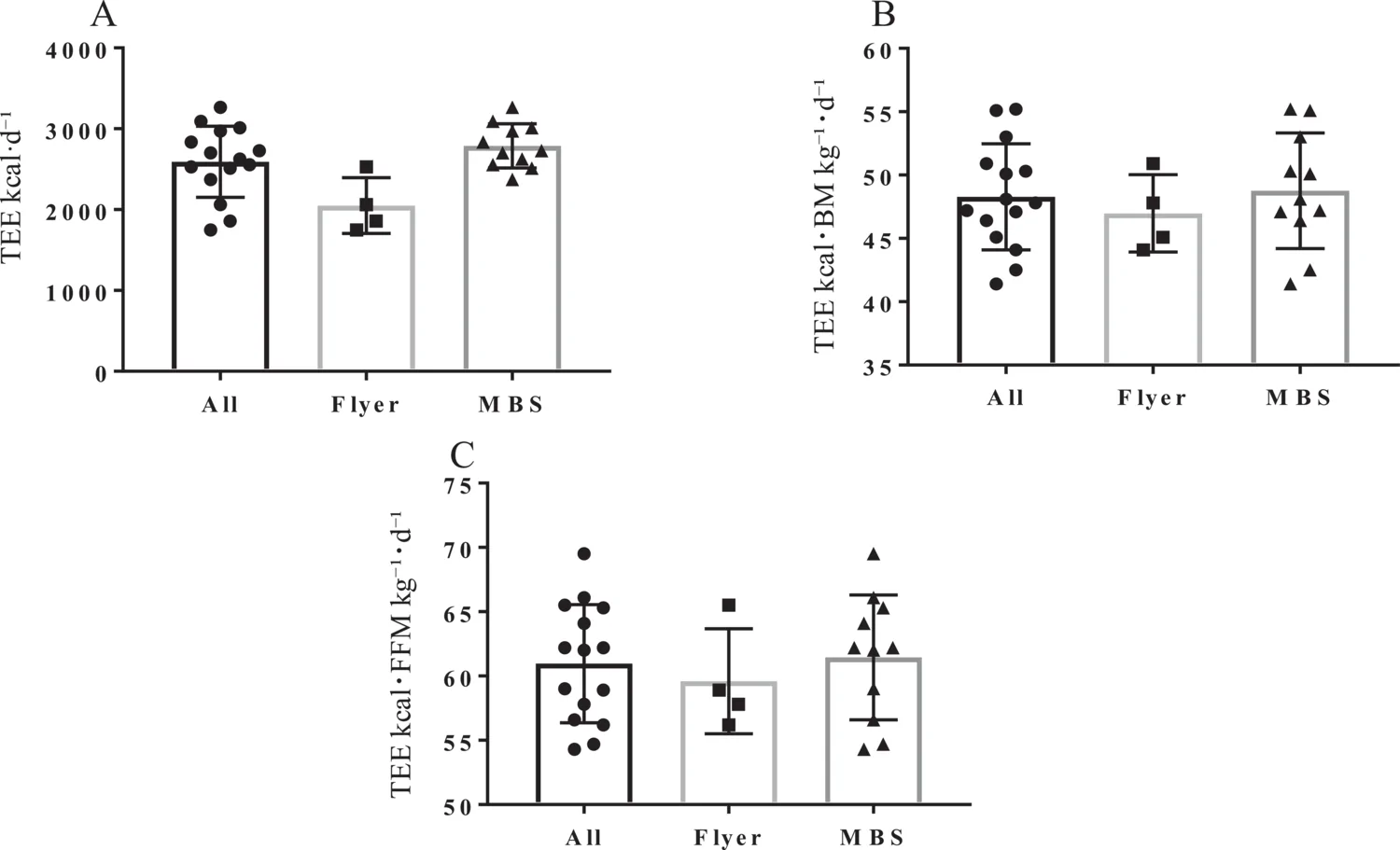
Individual and mean values of total energy expenditure by playing position.(A) Mean total energy expenditure (TEE) over the 7-day measurement period expressed as kcal·d⁻¹.(B) Mean total energy expenditure normalized to body mass (kcal·kg⁻¹ body mass·d⁻¹).(C) Mean total energy expenditure normalized to fat-free mass (kcal·kg⁻¹ fat-free mass·d⁻¹).Bars represent mean ± standard deviation, and individual data points are shown for all participants (All, n = 15), flyers (n = 4), and MBS (n = 11). MBS indicates middle (side base), base, and spotter positions.

### 3.2. Energy intake and macronutrient intake

The average TEI over the 3-day period was 1,873 ± 520 kcal·d^−1^ (range: 1,748–3,268 kcal·d^−1^), corresponding to 35 ± 9 kcal·kg^−1^ BM·d^−1^ (range: 18.1–50.7 kcal·kg^−1^ BM·d^−1^). Macronutrient intake was 75.9 ± 23.7 g·d^−1^ (1.4 ± 0.5 g·kg^−1^ BM·d^−1^) for protein, 65.8 ± 25.0 g·d^−1^ (1.2 ± 0.5 g·kg^−1^ BM·d^−1^) for fat, and 260.0 ± 65.2 g·d^−1^ (4.9 ± 1.1 g·kg^−1^ BM·d^−1^) for carbohydrate.

When expressed by positional group, protein intake was 1.4 ± 0.6 g·kg^−1^ BM·d^−1^ in flyers and 1.5 ± 0.4 g·kg^−1^ BM·d^−1^ in MBS, fat intake was 1.2 ± 0.5 and 1.3 ± 0.5 g·kg^−1^ BM·d^−1^, respectively, and carbohydrate intake was 4.3 ± 1.4 and 5.1 ± 1.0 g·kg^−1^ BM·d^−1^. Individual data points by positional group are shown in Figure 3.

**Figure 3.**
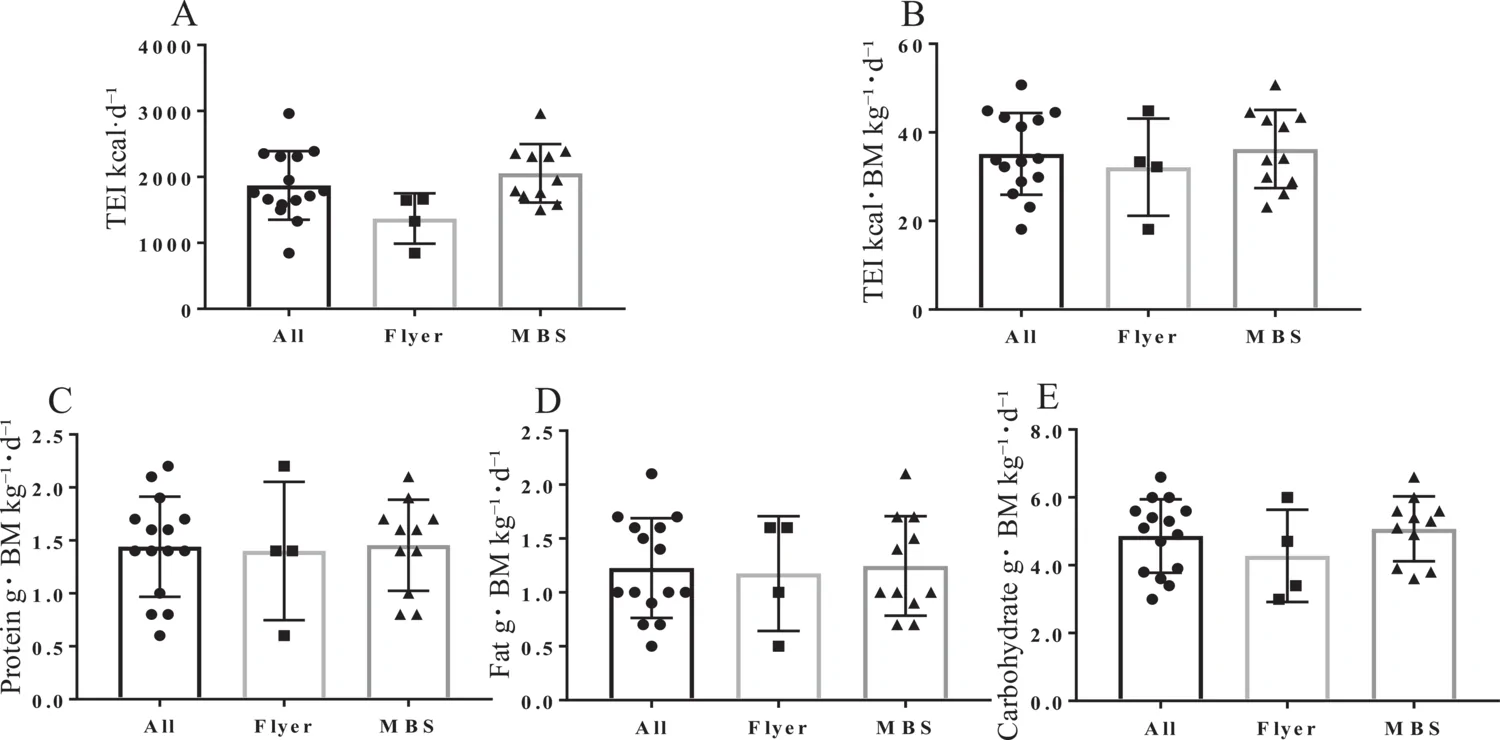
Individual and mean values of total energy and macronutrient intake by playing position.(A) Mean total energy intake (TEI) over the 3-day dietary assessment period expressed as kcal·d⁻¹.(B) Mean total energy intake normalized to body mass (kcal·kg⁻¹ body mass·d⁻¹).(C) Mean protein intake (g·d⁻¹).(D) Mean fat intake (g·d⁻¹).(E) Mean carbohydrate intake (g·d⁻¹).Bars represent mean ± standard deviation, and individual data points are shown for all participants (All, n = 15), flyers (n = 4), and MBS (n = 11). MBS indicates middle (side base), base, and spotter positions.

### 3.3. Relationships between energy expenditure, EI, and body composition

A significant positive relationship was found between the 7-day TEE and BM (Figure 4). Furthermore, significant positive correlations were observed between TEE and BM (r = 0.993; *p* < 0.001) and between TEE and FFM (r = 0.995; *p* < 0.001).

**Figure 4.**
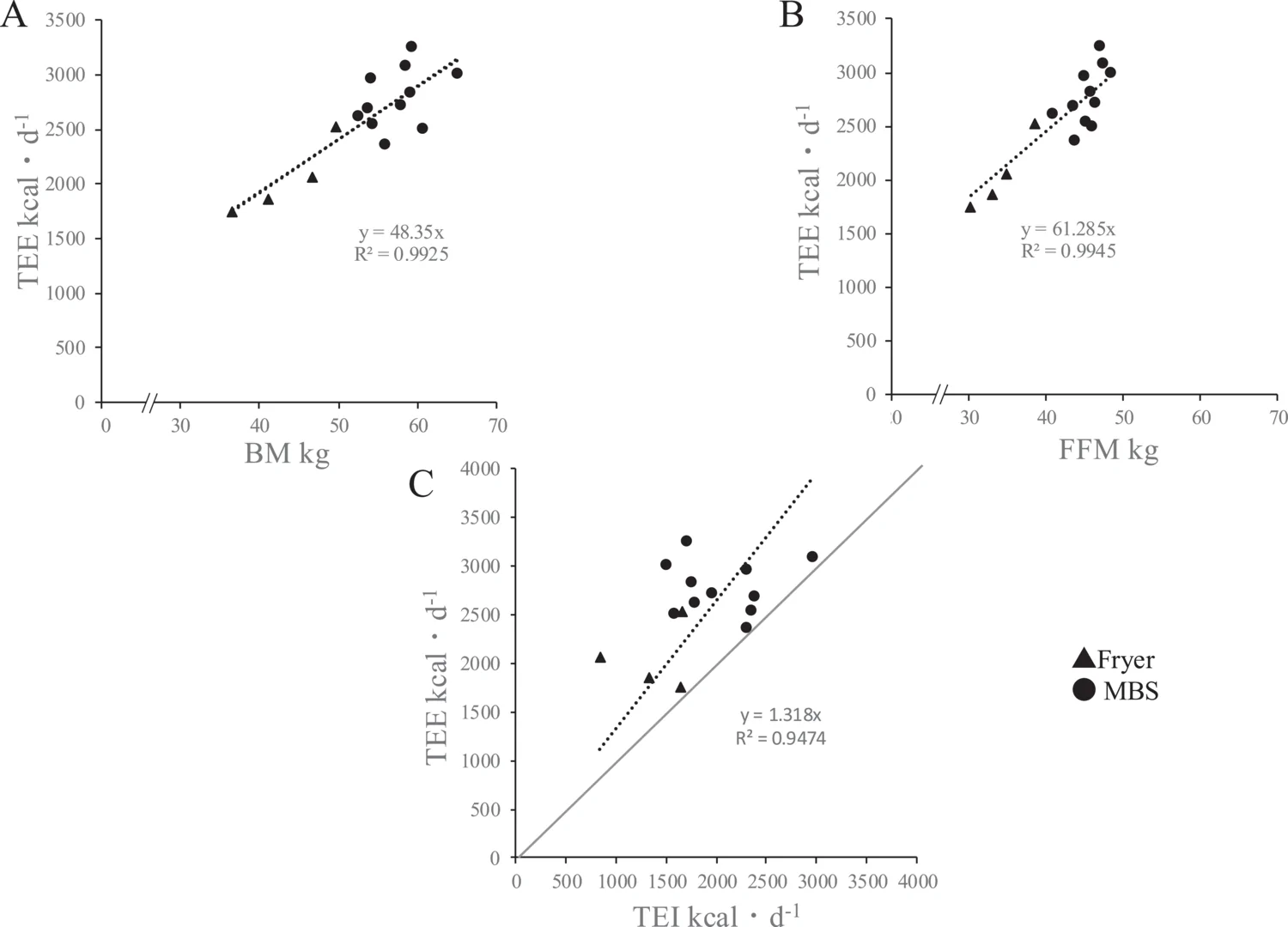
Relationships between total energy expenditure and body mass, fat-free mass, and energy intake.Scatter plots illustrating the relationships between mean 7-day total energy expenditure (TEE) and (A) body mass (BM), (B) fat-free mass (FFM), and (C) mean total energy intake (TEI). Linear regression lines are shown for descriptive purposes. In panel C, the solid line represents the line of identity (TEE = TEI). Flyers and MBS athletes are indicated by different symbols. MBS indicates middle (side base), base, and spotter positions.

On average, the self-reported EI was 27.3 ± 17.8% lower than the measured TEE (*p* < 0.05).

## 4. Discussion

To our knowledge, this is the first study to directly measure the TEE in elite female competitive cheerleaders using the DLW method. The present study demonstrated a high TEE (2,591 ± 440 kcal･d^−1^) and a mean PAL of 2.21 ± 0.17 over a 7-day in-season period. Previous assessments of daily TEE in female cheerleaders have relied on indirect methods, such as Polar Ignite watches, heart rate monitors, activity logs, and prediction equations (2, 25). These studies reported the TEE of professional cheerleaders to be 2,468 ± 427 kcal･d^−1^ and 2,199 ± 360 kcal･d^−1^, respectively [2,25]. The daily TEE obtained in the present study using the DLW method fell within the range of values previously reported in female cheerleaders using indirect methods. Competitive cheerleading involves explosive movements, including tumbling, stunting, pyramids, and partner lifting, which impose substantial physical demands on athletes [2,26]. These sport-specific characteristics likely contribute to the relatively high TEE observed in the present study despite the intermittent nature of the activity. Furthermore, the TEE observed in the present study was comparable to values reported in athletes participating in other high-intensity sports [12,13], further supporting the substantial physiological demands of competitive cheerleading. Accordingly, the present findings emphasize the need for appropriate nutritional strategies to support training and recovery in elite competitive cheerleaders.

The PAL data further support the high daily physical activity load observed in our study. The mean PAL in the present study was 2.21 ± 0.17 (range, 1.97–2.52). PAL data derived from the DLW method in Asian female athletes showed mean PAL values of 1.97 for collegiate tennis players, 2.18 for Japanese synchronized swimming athletes, and 2.68 for collegiate endurance runners [20,27,28] These variations likely reflect differences in training volume, sport-specific demands, and competitive level. Because PAL reflects habitual physical activity relative to resting metabolic rate, the relatively high PAL observed in the present study is consistent with a high overall daily physical activity load in elite competitive cheerleaders. In Western female athletes, mean PAL values were 1.71 for collegiate swimmers, 1.79 for international soccer players, and 2.1 ± 0.2 and 1.99 for national-level endurance runners during their training period, and 2.24 for cross-country runners [12,13,27,29]. These comparisons suggest that the PAL observed in the present study falls within the range reported for other highly trained female athletes despite differences in sport-specific demands. Therefore, PAL may provide a useful index for estimating energy requirements in competitive cheerleaders beyond training duration alone.

TEI during the 7-day assessment period was 1,873 ± 520 kcal·d^−1^, approximately 27% lower than the measured TEE. Because TEI and TEE were assessed using different methods (dietary intake records vs. DLW), this difference should not be interpreted as a precise estimate of energy balance. The dietary records method was used primarily to evaluate nutrient intake. Regarding energy intake, it was treated as a reference value, considering the potential influence of underreporting inherent to self-reported dietary assessment methods. Even considering the possibility of underreporting, the consistently lower reported TEI relative to measured TEE suggests that some athletes may not habitually consume sufficient energy to match their daily expenditure. This discrepancy may have practical implications because prolonged inadequate energy intake could impair recovery, training adaptation, and long-term health. Rather than providing an exact measure of energy balance, these results offer a descriptive indication that several participants may have had lower TEI compared with their measured TEE. In comparison with established recommendations, protein intake in this study fell within the recommended range of 1.2–2.0 g･ kg^−1^ BM for athletes [30]. Fat intake, expressed relative to BM, was slightly higher than the typical proportion of 20–35% of total energy recommended by the American College of Sports Medicine (ACSM) nutrition guidelines [31] Carbohydrate intake (4.9 g･kg^−1^ BM) was below the recommended 5–7 g･kg^−1^ BM for moderate training and 7–10 g･ kg^−1^ BM for high-intensity training [27,28], suggesting that the athletes may be at risk of insufficient carbohydrate availability to optimize performance and recovery. Because competitive cheerleading shares characteristics with other esthetic and weight-sensitive sports, such as gymnastics, athletes may also be susceptible to low energy availability and its associated health and performance consequences. Importantly, these findings highlight the need for further investigation of EA and RED-S-related health and performance outcomes in competitive cheerleaders [32,33].

While the relationship between TEI and TEE can offer insight into dietary adequacy, discrepancies between methods (DLW vs. self-reported dietary intake records) should be interpreted cautiously.Energy deficiency caused by unintentional undereating has been reported among cheerleaders [34]; furthermore, in a previous study, all cheerleaders exhibited low energy intake on practice days, with more than half showing signs of being at risk of eating disorders [2]. Importantly, dietary recording methods tend to underestimate energy intake. For example, DLW-based studies have shown that EI estimated from dietary records tends to be underreported in athletes [4] In a sample of Italian collegiate team-sport athletes, 56% were classified as low-energy reporters [5], and 61% of elite female gymnasts underreported their EI [6]. Although the mechanisms underlying underreporting remain unclear, it appears more common among women—possibly due to gender-related factors—and is associated with higher BM index, greater body fat, and lower TEI [5]. Collectively, these findings underscore the importance of considering both the potential risk of negative energy balance and the limitations inherent in dietary recording methods when evaluating the nutritional status of female athletes.

Although energy availability was not calculated in the present study, recent evidence indicates that calculated EA values should be interpreted cautiously because of methodological limitations in assessing its components and substantial interindividual variability in the EA levels associated with health and performance outcomes [32,35]. Therefore, future studies should combine objectively measured TEE with assessments of dietary intake, physiological function, athlete condition, and performance outcomes to better understand the impact of inadequate energy intake in competitive cheerleaders.

DLW - derived TEE provides an objective measure of free-living energy expenditure and can serve as a basis for estimating athletes’ energy requirements. However, TEE alone does not indicate whether energy intake is sufficient to support physiological function and health, particularly in athletes experiencing chronic energy deficiency [36]. These findings highlight the importance of educating competitive cheerleaders and their support staff about adequate energy and nutrient intake to meet the high energy demands of training and to support health, recovery, and performance.

Overall, the present study provides the first objective DLW-based assessment of TEE and PAL in elite female competitive cheerleaders, establishing an objective basis for estimating energy requirements in this population. The present findings also provide a foundation for future studies investigating EA, athlete condition, performance outcomes, and sport-specific nutritional strategies, ultimately contributing to evidence-based nutritional support for competitive cheerleaders.

## 5. Limitations

This study has some limitations. First, the sample size was relatively small (*n* = 15) and consisted exclusively of elite female competitive cheerleaders from a single team. This may limit the generalizability of the findings to other cheerleaders of different skill levels, ages, sexes, or cultural backgrounds. Second, the measurement period was limited to a 7-day in-season timeframe during the moderate training period, which may not reflect variations in TEE and dietary intake across different training phases or during the off-season. In particular, during the 1-month period before the competition, as training time, frequency, and intensity increase, higher TEE values would likely be observed than those reported in the present study. Finally, although the DLW method is the gold standard for measuring TEE, it does not provide detailed information on the specific components of energy expenditure or the types of physical activities contributing to it. Future studies should aim to combine DLW-based TEE measurements with objective assessments of training load to better characterize the determinants of total daily energy expenditure.

## 6. Conclusions

In summary, this study provides the first direct assessment of TEE in elite female competitive cheerleaders using the DLW method. The high TEE and PAL observed in this study highlight the substantial energy demands of competitive cheerleading. These findings provide objective reference data for estimating energy requirements and may assist sports dietitians, coaches, and clinicians in developing nutritional strategies to support adequate energy and nutrient intake, recovery, health, and performance in elite competitive cheerleading.

## Supplementary Material

Supplementary_Table_S1.pdf

SUPPORTING_INFORMATION.docx
