# Supplementary material for: Total energy expenditure of elite female cheerleaders assessed by doubly labeled water
Source: J Int Soc Sports Nutr. 2026 Nov 11;23(1):2733472. doi: 10.1080/15502783.2026.2733472 (PMC13761035; doi:10.1080/15502783.2026.2733472)
Supplement: Supplementary file 1 [file RSSN_A_2733472_SM2422.pdf]

Supplementary Table S1. Weekly training schedule during the 7-day measurement period

Training schedule

	day0	day1	day2	day3	day4	day5	day6
	Friday	Saturday	Sunday	Monday	Tuesday	Wednesday	Thursday
	Training	Training	Rest day	Rest day	Training	Training	Training
Team practice<br>・warm-up<br>・dance<br>・stunts<br>・tumbling<br>・cooldown	3h	6.5h			2h	3.5h	3.75h

The figure illustrates the weekly team training schedule during the 7-day doubly labeled water measurement period. Team practice sessions included warm-up, dance, stunts, tumbling, and cooldown activities. Scheduled rest days occurred on day 2 and day 3, during which no team training was performed.

In addition to team-based practice, athletes also engaged in individually conducted training. Independent training sessions focusing on dance, stunts, and tumbling were performed for approximately 1.5–2.0 h per day, 3–4 times per week. Athletes in middle (side base), base, and back spotter positions additionally performed resistance training for approximately 1.5 h per day, 3–4 times per week. Flyers performed self-directed training consisting of aerobic exercise and circuit training for approximately 1.5 h per session, twice per week.
