# Supplementary material for: Total energy expenditure of elite female cheerleaders assessed by doubly labeled water
Source: J Int Soc Sports Nutr. 2026 Nov 11;23(1):2733472. doi: 10.1080/15502783.2026.2733472 (PMC13761035; doi:10.1080/15502783.2026.2733472)
Supplement: Supplementary file 2 [file RSSN_A_2733472_SM0878.docx]

**SUPPORTING INFORMATION**

**Training During the Measurement Period**

During the measurement period, participants engaged in both team-based practice sessions and position-specific individual training. Supplementary Table S1 provides an overview of the typical weekly training schedule during the study period.

**Independent Training (dance, stunts, tumbling)**

Participants performed self-directed training sessions lasting approximately 1.5–2.0 h per day, 3–4 times per week. These sessions primarily focused on improving individual dance, stunt, and tumbling skills, with structured activities aimed at enhancing technical proficiency, strength, and overall performance.

**Weight Training (Middle, Base, Back Spotter)**

Athletes fulfilling the roles of middle (side base), base, or back spotter completed self-guided resistance training sessions lasting approximately 1.5 h per day, 3–4 times per week. The training emphasized the development of muscular strength, stability, and endurance required to support complex athletic movements and role-specific demands within the team.

**Flyer Training (Flyers)**

Flyers participated in training sessions that combined aerobic exercise and circuit training, with each session lasting approximately 1.5 h, conducted twice per week. This training regimen was designed to improve cardiovascular fitness, muscular endurance, and overall agility, which are essential for executing acrobatic maneuvers and maintaining performance quality.
